# Modelling Fatigue Crack Growth in High-Density Polyethylene and Acrylonitrile Butadiene Styrene Polymers

**DOI:** 10.3390/polym16091299

**Published:** 2024-05-06

**Authors:** Rhys Jones, Anthony J. Kinloch, Andrew S. M. Ang

**Affiliations:** 1 Department of Mechanical and Aerospace Engineering, Monash University, Clayton, Melbourne, VIC 3800, Australia; 2 ARC Industrial Transformation Training Centre on Surface Engineering for Advanced Materials, School of Engineering, Swinburne University of Technology, John Street, Hawthorn, VIC 3122, Australia; aang@swin.edu.au; 3 Department of Mechanical Engineering, Imperial, Exhibition Road, London SW7 2AZ, UK; a.kinloch@imperial.ac.uk

**Keywords:** acrylonitrile butadiene styrene, high-density polyethylene, fatigue crack growth, *R* ratio, simple-scaling, Hartman–Schijve equation

## Abstract

Prior studies into fatigue crack growth (FCG) in fibre-reinforced polymer composites have shown that the two methodologies of Simple-Scaling and the Hartman–Schijve crack growth equation, which is based on relating the FCG rate to the Schwalbe crack driving force, Δ*κ*, were able to account for differences observed in the measured delamination growth curves. The present paper reveals that these two approaches are also able to account for differences seen in plots of the rate of crack growth, *da/dt*, versus the range of the imposed stress intensity factor, Δ*K*, associated with fatigue tests on different grades of high-density polyethylene (HDPE) polymers, before and after electron-beam irradiation, and for tests conducted at different *R* ratios. Also, these studies are successfully extended to consider FCG in an acrylonitrile butadiene styrene (ABS) polymer that is processed using both conventional injection moulding and additive-manufactured (AM) 3D printing.

## 1. Introduction

For demanding applications, such as aerospace, automotive and pipelines, the service lifetime of HDPE is highly dependent on its resistance to the growth of small, naturally occurring cracks [1,2]. This aspect of its performance is of special significance, due to the possibility of internal and/or surface-breaking porosity or possibly the lack of fusion if the parts are produced using an AM process. As a result, United States Air Force Structures Bulletin EZ-SB-19-01 [3] states that all AM parts require a linear elastic fracture mechanics (LEFM) based durability and damage tolerance (DADT) assessment that is consistent with USAF MIL-STD-1530D [4]. Indeed, United States Air Force Structures Bulletin EZ-SB-19-01 [3] also states that the assessment and prediction of the durability and damage tolerance are perhaps the hardest challenges facing the structural integrity assessment of an AM part.

The Engineering Mechanics associated with fatigue crack growth in polymers and polymer blends, both conventionally and additively manufactured, has been active since the late 1960s [1,2,5,6,7,8,9,10,11,12,13,14,15,16,17,18,19,20,21,22,23,24,25,26], when it was first noted [5,9] that the rate of crack growth in a cycle, *da/dN*, could be related to Δ*K*, and the subsequent observation [6] that this may be the case for frequencies up to 100 Hz. An explanation of this phenomenon is given in [23], where it is also shown that the *S*-*N* curve, i.e., the stress–life curve, associated with polymers can be determined using linear elastic fracture mechanics (LEFM). (Here, *a* is the crack length, *N* is the cycle number, *S* is the applied stress amplitude, *da/dN* is the rate of crack growth per cycle, *K* is the crack tip stress intensity factor and Δ*K* is equal to *K_max_* − *K_min_*, where *K_max_* and *K_min_* are the maximum and the minimum values of the stress intensity factors in a load cycle, respectively). However, as can be seen from the recent reviews into slow crack growth in polymers [1,20], the equations used to model crack growth in polymers have not significantly changed from those reviewed by Radon in 1980 [12] and, as a result, are very similar to those used to assess crack growth in metals. It should also be noted that, as stated in ASTM test standard D7791 [27] and in [28], whilst a test frequency of between 1 and 25 Hz is allowable, a frequency of less than 5 Hz is recommended. This recommended lower test frequency of about 5 Hz is to reduce/minimise any possible effects due to heat generation.

The Nasgro crack growth equation [29]:

*da/dN* = *D*Δ*K_eff_*^(*m*−*p*)^ (Δ*K_eff_* − Δ*K_eff, thr_*)^*p*^/(1 − *K_max_*/*A*)^*q*^ (1)

This is perhaps the most commonly used crack growth equation that is commercially available in a software code. Here *D*, *m*, *p* and *q* are material constants, and *A* is the cyclic fracture toughness [29]. The term Δ*K_eff_* is the crack closure corrected value of Δ*K*, which for polymers is best expressed as originally suggested by Elber [30,31], that is:

Δ*K_eff_* = *U*(*R*) Δ*K* (2)

Here the *R* ratio is defined as *R* = *K_min_*/*K_max_* and the function *U*(*R*) is chosen such that, for long cracks that experience plastic wake-induced crack closure, the resultant *da/dN* versus Δ*K_eff_* curves, associated with each individual *R* ratio-dependent *da/dN* versus Δ*K* curve, all fell onto a single curve regardless of the *R* ratio. The term Δ*K_eff.thr_* in Equation (1) is the effective fatigue threshold, by this, we mean the value of Δ*K_eff_* at which *da/dN* = 0. Whilst, for metals, numerous closed-form expressions for *U*(*R*) have been suggested [32,33,34,35,36], it is unclear if any of these formulae hold for polymers [21]. As such, for polymers, it may be necessary to determine the function *U*(*R*) empirically from the measured *da/dN* versus Δ*K* data.

The studies and analyses into LEFM approaches to modelling crack growth in polymers discussed above have several shortcomings. For example, although it has long been known [37] that the *da/dN* versus Δ*K* curves associated with the growth of long cracks in metals can have a large variability, there is no comparable study into the variability of the *da/dN* versus Δ*K* curves associated with crack growth in polymers. The importance of accounting for this variability in design is highlighted in the NASA Fracture Control Handbook NASA-HDBK-5010 [38] that mandates the use of the worst case (i.e., mean-3σ) *da/dN* versus Δ*K* curve, where σ is the standard deviation. Furthermore, to date, fatigue tests on polymers have focused on the use of specimen geometries that are either as outlined in the main body of the fatigue test standard ASTM E647-15el [39] and have notch lengths of the order of 10 mm, or the crack round-bar test (CRB) specimen geometry [2,26] that have notch lengths of the order of 1 mm or greater. However, Section 5.1.5 and Appendix X3 in the ASTM fatigue test standard, ASTM E647-15el, explain that such tests do not reflect the *da/dN* versus Δ*K* curves seen in an operational structure, and as a result, designs based on these curves can be non-conservative. Appendix X3 also states that the analysis methods outlined in the main body of ASTM E647-15el are inappropriate. Overcoming these shortcomings requires tests on small naturally occurring cracks in polymers that enable the worst case (mean-3σ) small-crack *da/dN* versus Δ*K* curves to be determined. (Indeed, the practical need to determine the *da/dN* versus Δ*K* curves associated with small naturally occurring cracks is highlighted in [40,41,42,43], albeit for metallic structures). For metals, one approach to achieving this goal is to etch the surface [43] or to create a regular array of small etch notches [44]. In this context, it should be noted that Appendix X3 of ASTM E647-15el also notes that it is unclear if a fatigue threshold exists for naturally occurring small cracks in metals. As a result, the crack growth history associated with small naturally occurring cracks often tends to be exponential [45,46,47,48,49]. It is unclear if this phenomenon, i.e., the exponential growth, will hold for small naturally occurring cracks in polymers.

Recognising the need to be able to model and predict crack growth in HDPE, the present paper focuses on two novel approaches that have recently been successfully employed for assessing delamination growth in fibre-reinforced polymer composites and crack growth in metals. The first approach is the Hartman–Schijve variant of the Nasgro crack growth equation [45] that has been widely used to study the growth of small (naturally occurring) cracks in a range of both conventionally and AM metals [43,44,45,46,49,50,51,52,53,54,55,56,57,58,59,60,61,62,63]. as well as delamination growth in fibre-reinforced polymer composites [64,65,66,67,68,69], and crack growth in epoxy polymeric adhesives [70,71], nano-composites [72], polymers [73] and plasma-sprayed refractory metals [74]. As first explained in [45], the Hartman–Schijve equation is a special form of Equation (1) and is obtained by setting *m* = *p* and *q* = *p*/2. The Hartman–Schijve equation is available for use in the commercially available finite-element computer programs ABAQUS^®^, ANSYS^®^ and NASTRAN^®^ via the Zencrack software add-in version 9.3-1 [75]. An example of its use to study crack growth in an adhesively bonded doubler under representative multi-axial flight loads is given in [76]. The second approach studied is a Simple-Scaling methodology that was first proposed for metals in [77]. This approach was subsequently extended to delamination growth in fibre-reinforced polymer composites in [68,69]. The Engineering Mechanics behind this approach is explained in [78], where it is suggested that there should be a link between the Hartman–Schijve equation and the Simple-Scaling approach.

The examples studied in this paper reveal that the Hartman–Schijve crack growth equation is also able to account for the differences in the crack growth rate, *da/dt*, versus Δ*K* curves seen in *R* = 0.1 fatigue tests on three different HDPE polymers, before and after electron-beam irradiation, and to also account for the *R* ratio effects seen in fatigue tests on a commercially available HDPE polymer. In the latter instance, it is also shown that the Hartman–Schijve equation is able to collapse the test data obtained by using two different test specimen geometries onto a single master curve. We also show that, in all of the examples studied, a Simple-Scaling methodology is also successful in analysing the FCG rate in the HDPE polymers.

## 2. Materials and Methods

### 2.1. The HDPE Polymers

The first data set examined was the *da/dt* versus Δ*K* curves for *R* = 0.1, as given by Cerpentier et al. [79,80], which is for three different HDPE polymers as a function of varying degrees of electron-beam irradiation, i.e., 50, 100 and 150 kGy. These cyclic fatigue tests were performed at room temperature, i.e., 23 °C, and at a frequency of 5 Hz. The notation used in [79,80] to characterise these various specimen tests is given in Table 1, where *M_n_*, *M_w_* and *M_z_* are the molecular-weight distributions and χ_v_ is the crystalline volume fraction.

The second data set analysed was the *da/dt* versus Δ*K* curves given by Pinter et al. [2] for a commercially available HDPE polymer tested at *R* ratios of 0.1, 0.3 and 0.5. The fatigue tests were conducted at 80 °C and a frequency of 10 Hz. (Here it should be noted that [2] stated, for commercial-in-confidence reasons, details of this particular material could not be given).

### 2.2. The ABS Polymer

The *da/dt* versus Δ*K* FCG curves at *R* = 0.1 given in [81] for an acrylonitrile butadiene styrene (ABS) (Terluran^®^ GP-35 from Ineos, London, UK) polymer, processed using conventional injection moulding or additive manufacturing, i.e., via 3D printing [82], were also examined.

### 2.3. Methods of Data Analysis

As previously mentioned, the various *da/dt* versus Δ*K* curves given in [2,79,80] for tests on conventionally HDPE polymer manufactured and the *da/dt* versus Δ*K* curves given in [81] for ABS polymers processed using injection moulding or 3D printing were analysed using both the Hartman–Schijve [45] and the Simple-Scaling methodologies discussed in [68,69].

#### 2.3.1. The Hartman–Schijve Methodology

As previously noted, the Hartman–Schijve crack growth equation [45], which as shown in [45], is a special case of the Nasgro crack growth equation and has been shown [43,44,45,46,49,50,51,52,53,54,55,56,57,58,59,60,61,62,63,64,65,66,67,68,69,70,71,72,73,74] to be able to accurately capture and model:

(i) the growth of both small and long cracks in a range of both conventionally and AM metals;
(ii) the growth of both small and long cracks in structural adhesives;
(iii) the effect of temperature on crack growth in structural adhesives;
(iv) the effect of adhesive thickness on crack growth in structural adhesives;
(v) crack growth in nano-composites;
(vi) crack growth in plasma sprayed metals;
(vii) delamination growth in fibre-reinforced polymer composites.

This is available in the commercial finite-element programs ABAQUS, NASTRAN and ANSYS via the Zencrack computer software [75]. Furthermore, this approach can also be used to model the growth of complex-shaped 3D cracks in structural adhesives [76]. Therefore, the Hartman–Schijve approach was also used to study crack growth in the present fatigue tests on the HDPE and ABS polymers.

The form of the Hartman–Schijve used in this study is as given in [45], that is:

*da/dt* = *D* (∆*κ*)^*p*^ (3)

where *a* is the crack length, *t* is time, *D* and *p* are material constants and ∆*κ* [83] is the Schwalbe crack driving force, that is:

∆*κ* = (∆*K* − ∆*K_thr_*)/√(1 − *K_max_*/*A*) (4)

The term ∆*K_thr_* is the fatigue threshold, by this, we mean the value of ∆*K* at which *da/dt* = 0 m/s. That said, a range of other non-LEFM-based models such as those described in [84,85,86] have also been recently suggested.

#### 2.3.2. The Simple-Scaling Methodology

The paper by Schönbauer et al. [77] was the first to reveal that for conventionally manufactured metals, the *R* ratio effect vanished if the term *da/dN* was expressed as a function of ∆*K*/∆*K_th_*, where ∆*K_th_* is the value of ∆*K* at which *da/dN*= 10^−10^ m/cycle. This concept was subsequently validated in [69], where it was also extended to the statement that for conventionally manufactured metals, the *R* ratio effect vanished if the term *da/dN* was expressed as a function of ∆*K*/∆*K_da/dN_*, where ∆*K_da/dN_* is the value of ∆*K* at a low value of *da/dN*. References [68,69] extended this concept to delamination FCG in composites.

The explanation for this observation was recently given in [78], where it was shown that, for those problems for which Elber’s crack closure formulation held true, the function *U(R)* could be written as follows:

*U*(*R*) = Δ*K_eff,th_*/Δ*K_th_*(*R*) (5)

where Δ*K_eff,th_* is the fatigue threshold associated with the effective stress intensity factor Δ*K_eff_*.

Consequently, although the studies presented in [68,69,77,78] only dealt with metals and fibre-reinforced polymer composites, in the present paper, we also examine if expressing *da/dt* as a function of ∆*K*/∆*K_da/dt_* will:

(i) collapse the *R* = 0.1 *da/dt* versus Δ*K* curves associated with the tests on these various HDPE polymers that have been irradiated;
(ii) collapse the *da/dt* versus Δ*K* curves at values of *R* = 0.1, 0.3 and 0.5 given in [2] for a commercially available HDPE;
(iii) collapse the *R* = 0.1 *da/dt* versus Δ*K* curves associated with the tests on the ABS polymer.

#### 2.3.3. The Relationship between ∆*K*/∆*K*_*da/dt*_ and ∆*κ*

Since [78] suggested, but did not show, that there should exist a correlation between ∆*K*/∆*K_da/dt_* and ∆*κ*, in the present study, particular attention will be given to plotting ∆*K*/∆*K_da/dt_* as a function of ∆*κ*.

## 3. Results and Discussion

### 3.1. Crack Growth in a Range of HDPE Polymers

**Figure 1:**
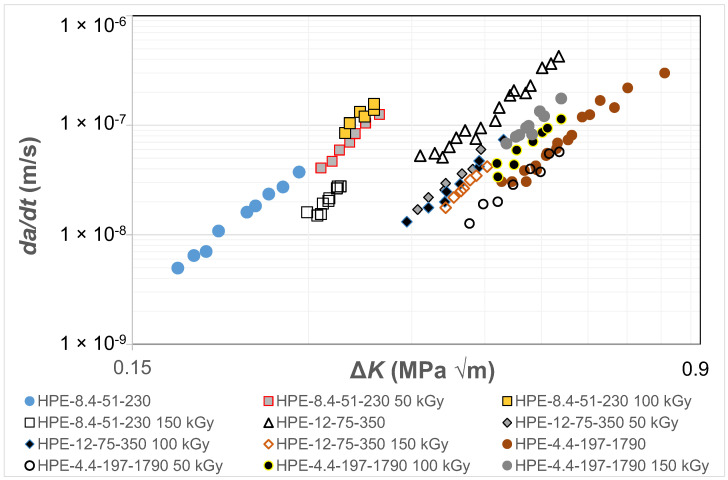
The effect of exposure to radiation on the *da/dt* versus Δ*K* curves for the HDPE polymers for fatigue tests at 23 °C and a frequency of 5 Hz.

The paper by Cerpentier et al. [79] presented the results for three different HDPE polymers, as a function of the degree of irradiation, at a fixed value of *R* = 0.1. The test frequency was 5 Hz so that, as noted in the ASTM test standard D7791 [27], heating effects should be minimal. The radiation levels to which these polymers were exposed were either 50, 100 or 150 kGy. The resultant *da/dt* versus Δ*K* curves associated with these 12 tests are shown in Figure 1. (Note that Figure 1 and all the subsequent fatigue plots use logarithmic axes). The data associated with tests on (non-irradiated) HPE-12-75-350 are augmented by additional data given in [80] for *R* = 0.1 tests on non-irradiated HPE-12-75-350. Figure 1 reveals that the different polymers have different *da/dt* versus Δ*K* curves and that exposure to radiation resulted in noticeable changes in the *da/dt* versus Δ*K* curves.

Cerpentier et al. [79,80] pointed out that HDPE is a semi-crystalline thermoplastic polymer, and electron-beam irradiation causes cross-linking of the polymer chains. The bulk of the cross-linking caused by the irradiation takes place in the amorphous phase, as opposed to the crystalline phase. Furthermore, although HDPE predominantly tends to cross-link upon irradiation, chain scission also occurs to a certain extent; again this tends to occur mainly in the amorphous phase. The results shown in Figure 1 reveal that the data can be described by an expression of the form of Equation (3) with an exponent that is independent of the degree of irradiation. This implies that the irradiation solely influences the pre-factor, *D*. Indeed, Cerpentier et al. [79,80] correlated the values of the pre-factor with various molecular structural parameters, i.e., *M_n_*, *M_w_* and *M_z_* and χ_v_, given in Table 1. The reader is referred to references [79,80] for further details, which are beyond the scope of the present paper.

**Figure 2:**
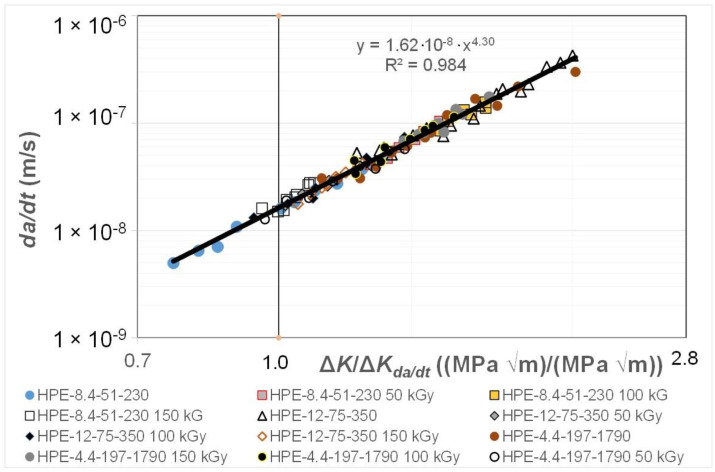
The fatigue crack growth curve for the HDPE polymers with *da/dt* expressed as a function of Δ*K*/Δ*K_da/dt_*.

**Figure 3:**
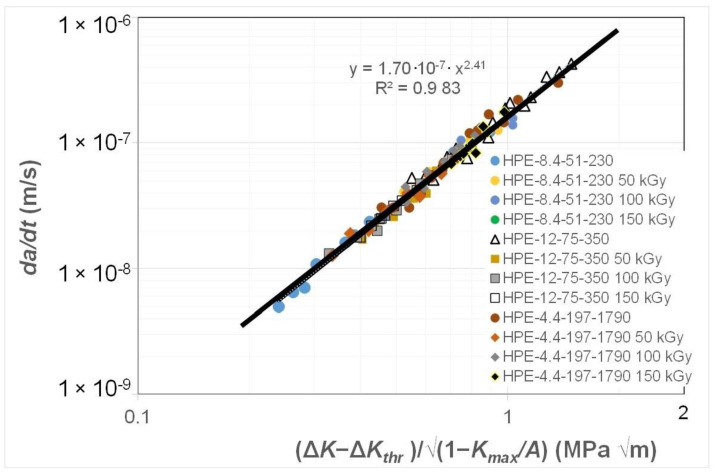
The fatigue crack growth curve for the HDPE polymers with *da/dt* expressed as a function of Δ*κ*.

**Figure 4:**
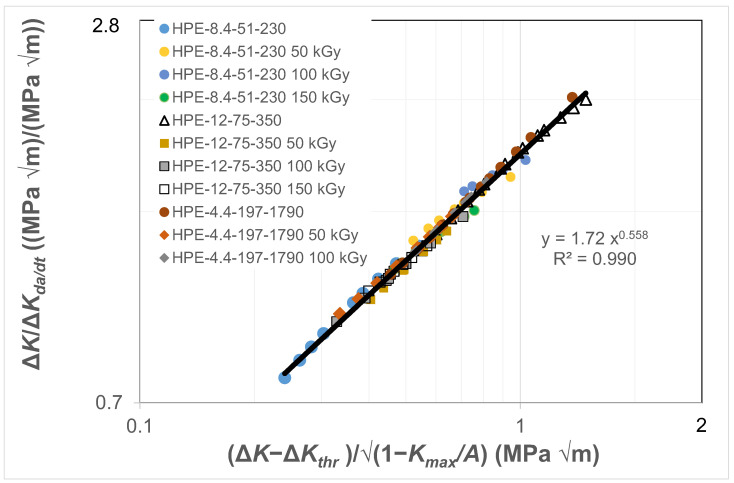
The relationship between Δ*K*/Δ*K_da/dt_* and Δ*κ* for the HDPE polymers.

Figure 2 and Figure 3 present the data shown in Figure 1 replotted with (a) *da/dt* expressed as a function of Δ*K*/Δ*K_da/dt_*, where the value of was chosen to correspond to a crack growth rate of 1.5 × 10^−8^ m/cycle, and (b) with *da/dt* expressed as a function of Δ*κ*. Figure 2 reveals that when *da/dt* is expressed as a function of Δ*K*/Δ*K_da/dt_*, the curves collapse onto what is essentially a single curve. Similarly, Figure 3 reveals that when *da/dt* is expressed as a function of Δ*κ*, the curves collapse onto what is essentially a single, albeit different, curve. In other words, the differences in the *da/dt* versus Δ*K* curves seen in Figure 1 vanish when account is made for the different values of the fatigue thresholds and the (cyclic) fracture toughness. Furthermore, Figure 4 reveals that, in these examples, there is a near-unique, strong correlation, with a coefficient of determination, R^2^, of 0.98 between Δ*K*/Δ*K_da/dt_* and Δ*κ*.

This observation suggests that the engineering mechanics underpinning the growth of cracks in polymers mirrors those explained in [78] for crack growth in metals in that, from a mechanics perspective, fatigue crack growth is largely controlled by the term Δ*K_da/dt_*. This is an interesting observation and further confirms that both the Hartman–Schijve [45] and the Simple-Scaling [68,69] methodologies would appear to be equally valid approaches that one can adopt to analyse the FCG rate data.

### 3.2. Crack Growth in an HDPE Tested at a Range of R Ratios

**Figure 5:**
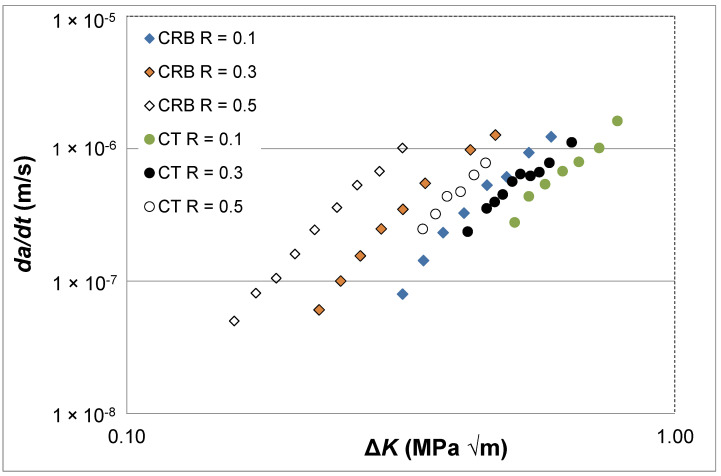
The *R* ratio and specimen dependency of the *da/dt* versus Δ*K* curves for fatigue tests using the commercially available HDPE polymer tested at 80 °C and a frequency of 10 Hz.

The above discussion has been confined to a single *R* ratio. However, for certification and quality control purposes, we also need to know how to characterise and model the *R* ratio dependency of the *da/dt* versus Δ*K* curves. Therefore, to continue the present study, let us examine the *da/dt* versus Δ*K* curves at *R* = 0.1, 0.3 and 0.5, as given in [2] for a commercially available HDPE polymer that was tested at 80 °C and a frequency of 10 Hz. (As per ASTM test standard D7791 [27] this means that heating effects are minimal). This study gave *da/dt* versus Δ*K* curves that were obtained using two different specimen test geometries. One of the specimen geometries used was the ASTM E647 compact tension (CT) specimen. The other test specimen geometry used was a circumferentially cracked round bar (CRB) specimen, see [2,26] for more details of the geometry of this particular test specimen. As can be seen in Figure 5, these two test configurations provided different *da/dt* versus Δ*K* curves, although it is unclear why these two different test geometries provided such different curves.

**Figure 6:**
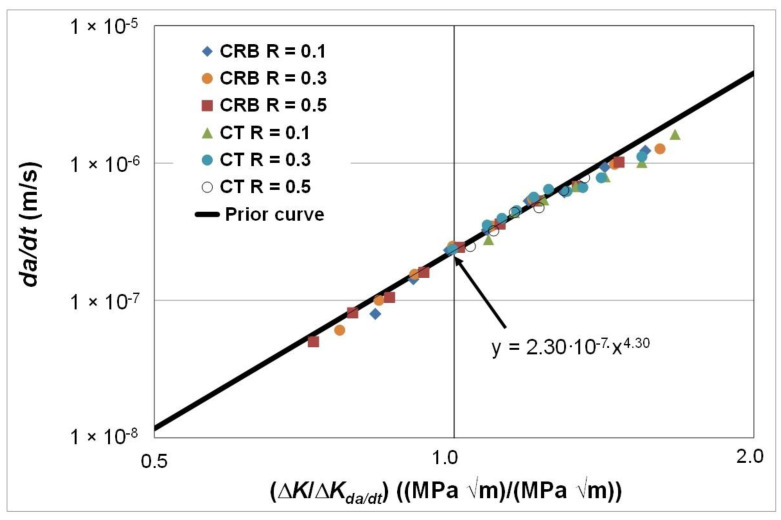
The *R* ratio and specimen dependency of the *da/dt* versus Δ*K*/Δ*K_da/dt_* curves for fatigue tests using the commercially available HDPE polymer tested at 80 °C and a frequency of 10 Hz.

As in the prior study, we first replotted these curves with *da/dt* expressed as a function of Δ*K*/Δ*K_da/dt_*. The resultant curves are shown in Figure 6. In this instance, the value of Δ*K_da/dt_* was chosen to correspond to a crack growth rate of approximately 2.3 × 10^−7^ m/s, see Figure 5. This value was chosen since it represented the lowest value of *da/dt* where all of the curves, shown in Figure 5, had a (near) common data point. Figure 6 also contains the prior curve predicted on the basis of the relationship between *da/dt* and Δ*K*/Δ*K_da/dt_* shown in Figure 2, i.e., the line of best fit shown in Figure 2, for the HDPE polymers studied in [79,80], that is:

*da/dt* = 1.62 × 10^−8^ (Δ*K*/Δ*K_da/dt_*)^4.3^ (6)

This was carried out by rescaling Equation (5) so that, when Δ*K*/Δ*K_da/dt_* = 1, the value of *da/dt* was equal to 2.3 × 10^−7^ m/s, see Figure 6. The resultant rescaled equation is as follows:

*da/dt* = 2.3 × 10^−7^ (Δ*K*/Δ*K_da/dt_*)^4.3^ (7)

The results shown in Figure 6 reveal that:

(i) Both the *R* ratio and the specimen test geometry dependency essentially vanish, i.e., the results fall onto a single curve, when *da/dt* is expressed as a function of Δ*K*/Δ*K_da/dt_*. As a result, the engineering mechanics behind this observation would appear to be as delineated in [78] for metals.
(ii) There is good agreement between this curve and the predicted curve that is based on tests discussed in Section 3.1 and shown in Figure 2.

In other words, for the various HDPE polymers studied, the relationship between *da/dt* and Δ*K*/Δ*K_da/dt_* is relatively independent of the nature of the polymer, the test geometry, the *R* ratio and the test temperature. As such this observation extends the engineering science given in [78] for conventional and additively manufactured metals to this class of problems.

**Figure 7:**
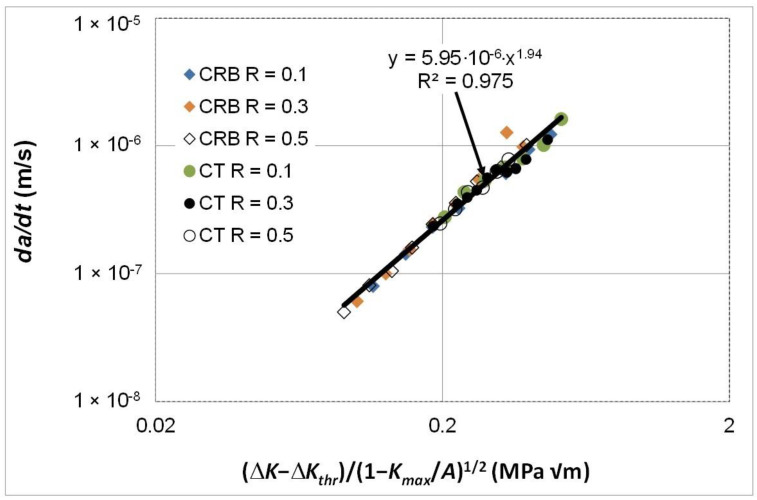
The *R* ratio and specimen dependency of the *da/dt* versus Δ*κ* curves for fatigue tests using the commercially available HDPE polymer tested at 80 °C and a frequency of 10 Hz.

The fatigue crack growth curves were next replotted with *da/dt* expressed as a function of Δ*κ*, and the resultant *da/dt* versus Δ*κ* curves are shown in Figure 7.

**Figure 8:**
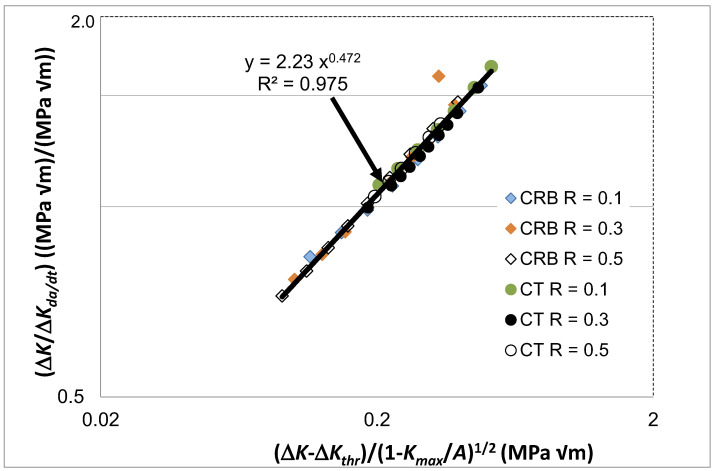
The relationship between Δ*K*/Δ*K_da/dt_* and Δ*κ* for fatigue tests using the commercially available HDPE polymer tested at 80 °C and a frequency of 10 Hz.

Figure 7 reveals that when *da/dt* is expressed as a function of Δ*κ* then both the *R* ratio and the specimen test geometry dependency disappear. In other words, the differences seen in the *da/dt* versus Δ*K* curves vanish when account is made for the different values of the fatigue thresholds and (cyclic) fracture toughness. Furthermore, as can be seen in Figure 8, it would appear that there is, again, a near-unique and strong correlation between Δ*K*/Δ*K_da/dt_* and Δ*κ*. Indeed, this relationship has a coefficient of determination, R^2^, of approximately 0.98. As previously noted, this finding reinforces the prior observation that the engineering mechanics underpinning the growth of cracks in polymers mirrors those explained in [78] for crack growth in metals. Thus, again, this interesting observation further confirms that both the Hartman–Schijve [45] and the Simple-Scaling [68,69] methodologies are equally valid approaches that one can adopt to analyse the FCG rate data.

### 3.3. Comparison of Results for the HDPE Polymers from [2,79,80]

Let us next compare the results from the three HDPE polymers, both non-irradiated and irradiated, reported in [79,80] to the one HDPE polymer reported in [2]. By examining Figure 2 and Figure 3 we understand that the FCG rate results for the three HDPE polymers, and for both the non-irradiated and irradiated materials, would (to a first approximation) appear to lie on a single, unique linear plot when the data are plotted on logarithmic scales as analysed using the Simple-Scaling, see Figure 2, or the Hartman–Schijve, see Figure 3, methodologies, respectively. This reflects the fact that, as commented in [79,80], relatively small differences were observed in the FCG kinetics as a function of irradiation dose, which is quite remarkable, since the molecular-weight distribution changes drastically with irradiation dose. Currently, the results for the FCG rate for the one HDPE polymer reported in [2], where the tests were undertaken as a function of the *R* ratio and the type of test specimen employed, are internally consistent. By this, we mean that, as shown in Figure 6, the test data lie on a single, unique linear plot when the data are plotted on logarithmic scales using the Simple-Scaling. Similarly, Figure 7 reveals that when the data are plotted as per the Hartman–Schijve formulation the data also lie on a single, unique linear plot. However, comparing Figure 4 and Figure 8, we find that the relationships between Δ*K*/Δ*K_da/dt_* and Δ*κ* differ. Unfortunately, since no details of the HDPE polymer used in the tests reported in [2] are given due to commercial confidentiality, no comments on the reasons for this observation are possible.

### 3.4. Crack Growth in Injection-Moulded and 3D-Printed ABS Polymer

**Figure 9:**
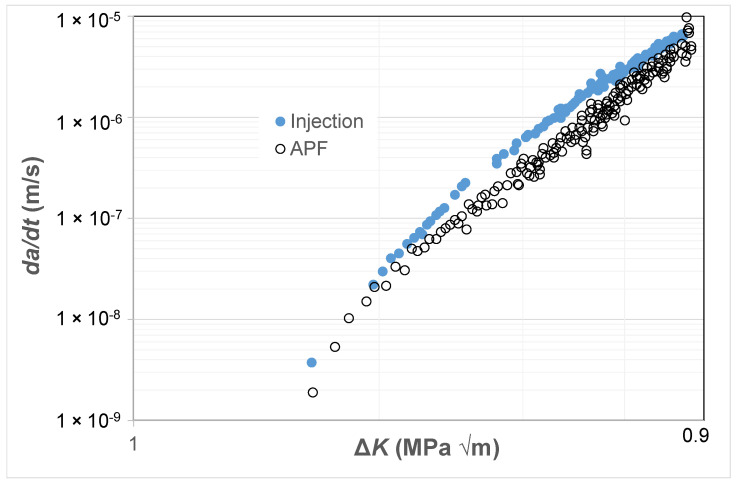
The *da/dt* versus Δ*K* curves for fatigue tests on the ABS test specimens manufactured via a conventional injection-moulding process or by an AM 3D-printing (i.e., the APF) process.

**Figure 10:**
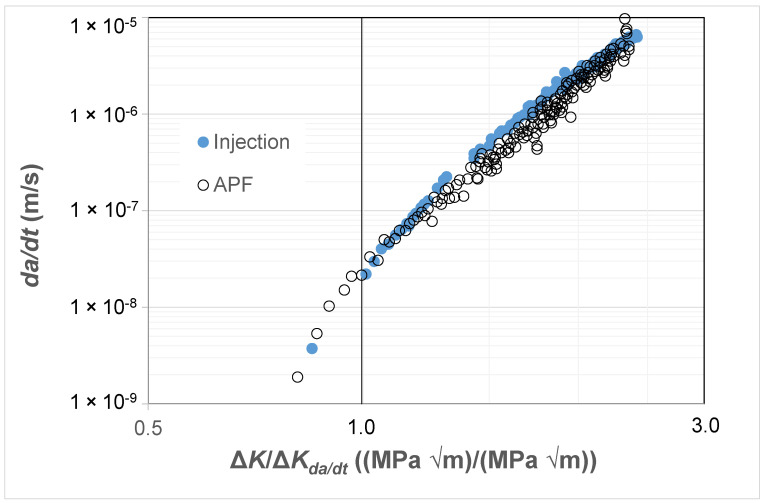
The *da/dt* versus Δ*K*/Δ*K_da/dt_* curves for tests on the ABS test specimens manufactured via a conventional injection-moulding process or by an AM 3D-printing (i.e., the APF) process.

**Figure 11:**
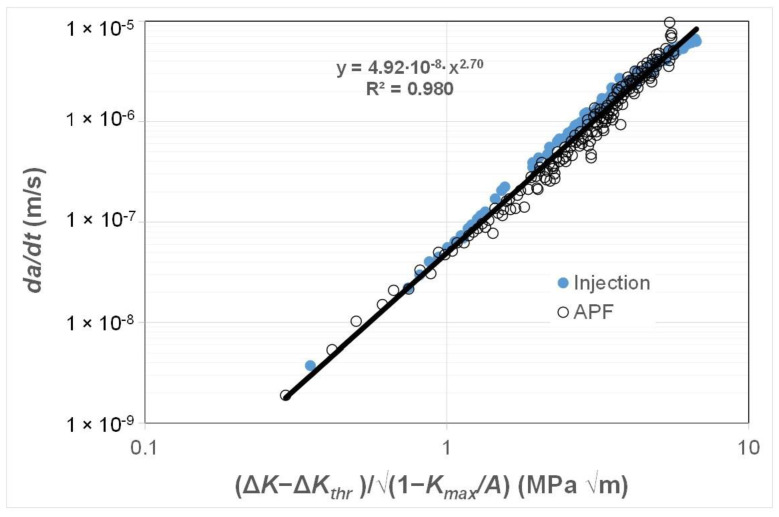
The *da/dt* versus Δ*κ* curves for fatigue tests on the ABS test specimens manufactured via a conventional injection-moulding process or by an AM 3D-printing (i.e., the APF) process.

As previously noted there are currently only a few studies that present fatigue crack growth curves associated with AM polymers and even fewer that present the FCG curves associated with the growth of small naturally occurring cracks in either AM or conventionally manufactured polymers. Consequently, to continue this study, we chose to examine the *R* = 0.1 *da/dt* versus Δ*K* curves given in [81] for an ABS (Terluran^®^ GP-35) polymer where the test specimens were manufactured using a conventional injection moulding or an AM 3D-printing process, more specifically by what is termed an “Arburg plastic free-forming (APF)” process and for more details see [81,82]. As can be seen in Figure 9, these two different manufacturing processes provided somewhat different *da/dt* versus Δ*K* curves. As in the prior studies, Figure 10 and Figure 11 present these curves replotted with *da/dt* expressed as a function of Δ*K*/Δ*K_da/dt_* and Δ*κ*, respectively. Figure 10 and Figure 11 both reveal that, when *da/dt* is expressed as a function of Δ*K*/Δ*K_da/dt_* or Δ*κ,* the experimental results associated with the injection-moulded and the 3D-printed specimens essentially collapse onto one unique relationship previously noted and the 3D-printed specimens essentially collapse onto one unique relationship. This observation further supports the belief that the engineering mechanics underpinning the growth of cracks in polymers mirrors those explained in [78] for crack growth in metals.

**Figure 12:**
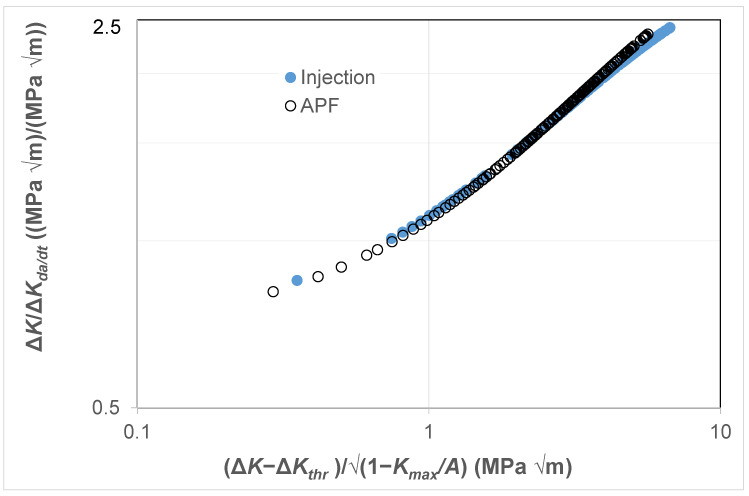
The relationship between Δ*K*/Δ*K_da/dt_* and Δ*κ* for fatigue tests on the ABS test specimens manufactured via a conventional injection-moulding process or by an AM 3D-printing (i.e., the APF) process.

Finally, as can be seen in Figure 12, it would appear that there is, again, a near-unique correlation between Δ*K*/Δ*K_da/dt_* and Δ*κ*. Thus, again, this interesting observation further reveals that the engineering science developed in [78] for crack growth in conventionally and additively manufactured metals appears to hold for crack growth in these polymers. Indeed, it should also be noted that [68,69] have shown that both the Hartman–Schijve and the Simple-Scaling methodologies are equally valid approaches that can be used to analyse fatigue crack growth in a range of fibre-reinforced polymer composites.

## 4. Conclusions

This paper has shown that the Engineering Mechanics that links the Simple-Scaling and the Hartman–Schijve crack growth equation for metals also holds for crack growth in tests on a range of HDPE polymers as well as in the two ABS polymers studied. As a result, as first suggested in [78] for conventionally and additively manufactured materials as well as for a medium entropy alloy, the function *U(R)* used to relate Δ*K* to Δ*K_eff_* for these various polymers would appear to be inversely proportional to Δ*K_da/dt_*. Furthermore, from a fracture mechanics perspective, for the various HDPE polymers studied, the relationship between *da/dt* and Δ*K*/Δ*K_da/dt_* would appear to be relatively independent of the nature of the polymer, as well as the degree of irradiation, the test geometry and the *R* ratio. This observation suggests that, from a fracture mechanics perspective, fatigue crack growth in these tests would appear to be largely controlled by the term Δ*K_da/dt_*. We also have shown that, for the polymers studied, there is a strong correlation between the ratio Δ*K*/Δ*K_da/dt_* and the Schwalbe crack driving force, Δ*κ.*

## Figures and Tables

**Table 1:** HDPE polymer descriptors from Cerpentier et al. [79,80].

Descriptor	*M_n_* (kDa)	*M_w_* (kDa)	*M_z_* (kDa)	χ_v_ [% v]	Dose (kGy)
HPE-12-75-350	12	75	350	74.4	0
HPE-8.4-51-230	8.4	51	230	71.4	0
HPE-4.4-197-1790	4.4	197	1790	74.2	0
HPE-12-75-350-50	7	120	880	75.2	50
HPE-8.4-51-230-50	9	74	610	71.8	50
HPE-4.4-197-1790-50	8	130	700	73.8	50
HPE-12-75-350-100	6	59	340	75.1	100
HPE-8.4-51-230-100	8	105	1000	71.2	100
HPE-4.4-197-1790-100	5	44	195	73.9	100
HPE-12-75-350-150	8	37	145	74.9	150
HPE-8.4-51-230-150	5	82	750	71	150
HPE-4.4-197-1790-150	3	21	77	73	150

## Data Availability

The data will be made available at the completion of the project.

## Nomenclature

*a*	total crack length, measured from the loading line
*A*	constant in the Hartman–Schijve equation
ABS	acrylonitrile butadiene styrene
AM	additive-manufactured
*da/dt*	rate of fatigue crack growth
CRB	cracked round-bar test
CT	compact tension test
*D*	a constant in the Hartman–Schijve and Nasgro crack growth equations
DADT	durability and damage tolerance
*m* and *q*	constants in the Nasgro crack growth equation
FCG	fatigue crack growth
HDPE	high-density polyethylene
K	stress intensity factor
$K_{max}$	maximum value of the applied stress intensity factor in the fatigue cycle
$K_{min}$	minimum value of the applied stress intensity factor in the fatigue cycle
ΔK	range of the applied stress intensity factor in the fatigue cycle, as defined below $∆K=K_{max}-{\;K}_{min}$
Δ*K_da/dt_*	the value of ΔK at a low value of *da/dt*
Δ*κ*	the Schwalbe crack driving force
LEFM	linear elastic fracture mechanics
NASA	North American Space Administration
NIST	National Institute of Standards and Technology
*p*	exponent in the Hartman–Schijve and Nasgro crack growth equations
*P_max_*	maximum load applied during the fatigue test
*P_min_*	minimum load applied during the fatigue test
R	load ratio (=*P_min_/P_max_*)
RAAF	Royal Australian Air Force
R^2^	coefficient of determination
*t*	time
*S*	applied stress amplitude
USAF	United States Air Force
